# Woman’s Helper

**Published:** 1870-11

**Authors:** 


### WOMAN'S HELPER.

The greatest material and moral helper of woman in all ages, is A. T. Stewart—not only the greatest in pecuniary extent, but the greatest in wisdom, for it is the embodiment of a true philanthropy. He is expending three millions of dollars in order to help the helpless who are willing to help themselves; that is, any poor unmarried woman who has no one to look to for aid, and is willing to work for herself, in this crushing, crowding New York city. The help will be offered in this way—a nice, tidy, cosy, well lighted, well warmed room will be furnished for one dollar a week. The washing and the food will not exceed two dollars a week.

The rent must always be paid in advance; this prevents going in debt, and the demoralizing effect of incurring an obligation with the hap-hazard, trusting-to-luck for the wherewith to meet the engagement, and, if not supplied, the temptation to false promises, frivolous excuses, and sneaking away in the night.

Every room will be fitted up with comparative elegance, comfortably warmed and lighted, and must be kept in perfect order, tidiness, and cleanliness by the occupant, and in default, immediate dismissal will follow. With such surroundings, self-respect will be maintained, and moral elevation will be a necessary result.

The food will be the very best, and prepared by the most experienced and skilled cooks. A list of the articles prepared, with the cost of each attached, will be furnished so that each one will pay only for what is ordered, the cost being a dime, or a dollar or more a day, according to the choice of those calling for a meal. A cup of coffee will not exceed two cents, a plate of meat five cents, a dish of vegetables three, and all other things in proportion.

The entire building and ground costs three millions of dollars, and will accommodate fifteen hundred persons. Most of the rooms will be single, eight feet by nine; but in case of sisters, mother and daughter, some of the rooms will be double, eighteen feet by sixteen. There will be a library and reading room, and also a large hall for lectures and concerts, at a nominal cost.

Visiting and visitors will be allowed as at other hotels, without any requirements beyond those of propriety of deportment. The establishment is in no sense a work-house, or poor-house, or asylum; it is a hotel for women who pay for what they get, who can remain as long as they choose, leave when they like, and return when it suits them, with as much freedom as those who frequent the Fifth Avenue, the Astor, or the Grand Central. If a grander programme of philanthropy for unmarried women dependant for a living on their own personal efforts has ever been devised, it has not been brought to our attention.

The building is now in its third story, and its completion hoped for during 1872. The building will be seven stories above ground, covers forty-one thousand square feet, height one hundred and nine feet, built of iron and brick, and cannot burn down.

